# Sustainable Choices and Behaviors among Eco-Friendly Museum Travelers: Exploring the Drivers of Sacrifice, Visit, Pay, and WOM Intentions

**DOI:** 10.3390/ijerph18020845

**Published:** 2021-01-19

**Authors:** Heesup Han, Linda Heejung Lho, Hyeon-Cheol Kim, Elena-Nicoleta Untaru

**Affiliations:** 1 College of Hospitality and Tourism Management, Sejong University, 98 Gunja-Dong, Gwangjin-Gu, Seoul 143747, Korea; heesup@sejong.ac.kr (H.H.); heeelho@gmail.com (L.H.L.); 2 School of Business Administration, Chung-Ang University, 84 HeukSeok-Ro, DongJak-Gu, Seoul 06974, Korea; 3 Faculty of Economic Sciences and Business Administration, Transilvania University of Brașov, Colina Universității nr. 1, Corpul A, etajul III, 500068 Brașov, Romania; elena.untaru@unitbv.ro

**Keywords:** eco-friendly museums, pro-environmental intentions, customer-product relationship quality, feeling of pride, connectedness to nature, social pressure, reputation, ecological value

## Abstract

This research developed a sturdy theoretical framework that offers a better comprehension regarding customer approach intentions for eco-friendly museum products. Using a quantitative process, the apparent role of ecological value, connectedness to nature, social pressure, pro-environmental reputation was explored. Data quality testing demonstrated the validity of the construct measures. The critical mediating nature of customer-product relationship quality and feeling of pride was unveiled by conducting a structural analysis. In addition, the feeling of pride was a prominent factor determining sacrifice, visit, pay, and word of mouth (WOM) intentions. Social pressure played a major role in building relationship quality, whereas pro-environmental reputation was a key contributor to increase the feeling of pride. The model contained a strong prediction power for intentions. Results of this study contribute to enriching the extant knowledge regarding customer pro-environmental decision-making process, which is helpful for an eco-friendly museum and its success.

## 1. Introduction

The museums, along with the hospitality and tourism industry (e.g., museums, hotels, restaurants, airlines, resorts, cruises), are one of the major contributors to environmental contaminations [1,2,3]. Museums often generate harmful impacts on the environment through greenhouse gas emission, water pollution, soil contamination, and solid/food waste generation [2,4]. Facing the environmental issue is unavoidable for museum operators [3,4,5] as the museum industry has been constantly expanding together with the development of the tourism industry [6]. Greening museums is nowadays inevitable for all museum operators [4,7]. Patrons in the tourism marketplace are also ecologically conscious more than ever, showing increasing needs/wants for environmentally responsible products [8,9]. Given this, inducing visitors’ eco-friendly approach intentions/behaviors for a museum by practicing pro-environmental management and implementing green technology is becoming an essential goal for museum operations [2,3,5].

Eco-friendly museums indicate environmentally-friendly museum operations that are proactive in minimizing the harmful effect on the environment [1,7]. For eco-friendly museum operators, understanding various variables that elicit customer approach intentions and behaviors can be a vital requisite for the survival and success of their operations [5,7]. Many researches in environmental psychology and consumer behavior indicated that diverse cognitive variables drive approach behaviors for eco-friendly products/services [10,11,12,13,14]. In particular, researchers in these studies insisted the criticality of ecological value [15,16], connectedness to nature [12,17,18], social norm [10,19], reputation [20,21], and relationship quality [11,22] for explaining pro-environmental behaviors. Despite the importance of these concepts, an empirical endeavor that unearths the possible relations of the variables has been rarely made.

In addition, recent researches in environmental behavior identified the significant role of the emotional process, whose key constituent is the feeling of pride, in forming pro-environmental intentions [23,24,25,26]. Nevertheless, relatively minimal effort has been made to explore the influence of proud feeling on museum customer eco-friendly decision formation. Additionally, integrating this emotional concept into the framework for explaining customer green behavior in the museum context as well as in the overall tourism sector has not yet been revealed. Moreover, the role of customer-product relationship quality and feeling of pride as mediators has been scarcely uncovered in the environmentally responsible consumption situation. The role of customer-product relationship quality and feeling of pride as mediators will affect the museum visitors’ approach behavior intentions. Thus, the present study attempted to reveal the role of mediators, making an empirical research effort to fill the void of previous studies.

The main purpose of this research thus was to develop a theoretical framework that offers a clear understanding of the creation of customer eco-friendly approach behavioral intentions for environmentally responsible museum products. Especially, this research sought (1) to investigate the complex relationships among ecological value, connectedness to nature, social pressure, pro-environmental reputation, customer-product relationship quality, and feeling of pride in generating approach behavioral intentions (sacrifice, visit, pay, and word-of-mouth (WOM) intentions), (2) to explore the mediating influence of customer-product relationship quality and feeling of pride, and (3) to unearth the comparative importance of study constructs in identifying the approach intention factors. The literature review is presented in the following section. Subsequently, the research method and result sections are presented. The discussions and implications are provided in the last section of this study.

## 2. Literature Review

### 2.1. Ecological Value and Its Role

Ecological value is broadly believed as a meaningful construct in the environmental behavior literature [13,15,27]. Authors [16] and [28] described personal value as important criteria that one utilizes to make choices, to verify behaviors, and to assess others and events. According to [13], altruistic, egoistic, and ecological values are three major constituents of personal value. Of these three major constituents, ecological value is of criticality in explicating environmentally responsible human behaviors [13,15,29]. In the present research, ecological value refers to the personal criterion that customers use to make ecologically responsible decisions related to an eco-friendly tourism product. Ecological value is interchangeably used with biospheric value or environmental value. Customers with high ecological value place emphasis on the advantages/disadvantages for the ecosystem when making their purchase decisions and behaviors [16,27,29]. In the hospitality context, [15], in their empirical research, uncovered that ecological value is a crucial factor, inducing a better relationship with a green product and eliciting a positive intention for the product. Within the pro-environmental employee behavior framework, [30] also uncovered the role of moral value and injunctive moral norms. Their empirical evidence indicated that the congruence between these two variables significantly influences the affective process, encompassing a feeling of pride for environmentally friendly behaviors. Besides, according to [31], “people receive non-material benefits from ecosystems through spiritual enrichment, cognitive development, reflection, recreation, and aesthetic experience”. [13] concluded that there are four big causal variables in deciding environmentally significant behaviors—two of which are habit and contextual forces. Patrons may routinely choose environmentally friendly options, and patrons may choose environmentally friendly options due to external forces, such as community expectations. When a patron chooses to take environment/ecology friendly options, we can assume that ecological value has made one routinely choose an environment-friendly product and formed customer-product relationship quality, while fulfilling community expectation bring a feeling of pride for the ones meeting the expectation. In regard to the arguments described above, we hypothesized the followings:

**Hypothesis** **1 (H1).** *Ecological value has a positive effect on customer-product relationship quality*.

**Hypothesis** **2 (H2).** *Ecological value has a positive effect on the feeling of pride*.

### 2.2. Connectedness to Nature and Its Role

Connectedness to nature is a patron’s emotional connectivity to the natural environment [12]. Similarly, [32] explained linkage to nature as the degree to which a patron senses a part of the natural environment. According to [33] and [18], connectedness to nature along with environmental values and beliefs are crucial drivers of sustainable lifestyle as well as sustainable customer behaviors (e.g., recycling). Connectedness to nature is also linked to altruistic/pro-social behaviors and intimacy, which is a major facet of relationship quality [17]. More recently, in their empirical research, [12] provided evidence that adherence to nature has a significant impact on relationship quality factors (e.g., affinity with nature) and affective engagement to nature in explaining generating green purchase behaviors, reutilization, and recycling. A sturdier feeling of connection to nature is related to a higher possibility of preserving the environment and to a greater propensity to maintain environmentally responsible consumption behaviors [7]. Those individuals who feel deep connectedness to nature often show stronger ecological concern, more positive affect to eco-friendly products, stronger relationship closeness, and a higher likelihood to engage in pro-environmental consumption [12,17,18,33]. Besides, according to [34], pro-environmental behavior can be interpreted as pro-social behavior. As pro-social behavior is a behavior that benefits others, it often evokes a feeling of pride, and thus based on the evidence and assertion of the above-mentioned studies, we developed the following hypotheses:

**Hypothesis** **3 (H3).** *Connectedness to nature has a positive effect on customer-product relationship quality*.

**Hypothesis** **4 (H4).** *Connectedness to nature has a positive effect on the feeling of pride*.

### 2.3. Social Pressure for Pro-Environmental Choices and Its Role

Social pressure has been considered as a fundamental concept in social psychology and eco-friendly consumption behavior [10,19]. Social pressure refers to customers’ perceived level of pressure from their important others (e.g., family, friends, co-workers) when practicing a particular action (or making a particular choice) [19,35]. For instance, customers are likely to feel a certain level of pressure if they behave against their significant others’ beliefs (e.g., conduct environmentally irresponsible behaviors) [36]. According to [19], the person is likely to participate in a socially responsible activity when they feel high social pressure from others. Social pressure is hence utilized interchangeably with the term “subjective norm” or “social norm” [10]. Undeniably, this social pressure is a crucial contributor to eliciting customers’ environmentally responsible behaviors in a consumption situation [10,23,37]. In the tourism industry, [36] uncovered the positive relation between social pressure and anticipated feeling and identified the influence of such association on pro-environmental approach decision and relationship quality with a tourism product. In their meta-analytic research, [23] found that social pressure is strongly affiliated with the emotional process and relational bonds between a product and its patrons in the environmental behavior context. Thus, social pressure for pro-environmental choices may bring a positive effect on customer and product relational bonds and also affect customers’ emotions. Consistently, appertaining to the evidence of the above-mentioned researches, we developed the following hypotheses:

**Hypothesis** **5 (H5).** *Social pressure for pro-environmental choices has a positive effect on customer-product relationship quality*.

**Hypothesis** **6 (H6).** *Social pressure for pro-environmental choices has a positive effect on the feeling of pride.*

### 2.4. Pro-Environmental Reputation and Its Role

Product/service reputation is a key concept in consumer behavior and marketing [21,36,37,38]. Product reputation often forms based on one’s assessment of the long performance of the product and its attributes [39,40]. According to [21,37], product reputation is the set of cognitive perceptions that an individual has about a product, which has been built for a long time. Since this term is similar to the overall image of a product, product reputation image is recently used in an interchangeable manner [20]. In this research, pro-environmental reputation refers to the overall impressions that customers have about environment-friendly tourism commodities and their eco-friendly attributes, which have been formed for a long time. Product reputation is undoubtedly a major cognitive concept, influencing affective and conative processes in customer purchase decision formation and behaviors [39,40]. According to [21,40], based on product/service reputation built through their cognitive evaluation of the attractiveness of a specific product/service and the attractiveness of its attributes, consumers feel a certain degree of affect for the product/service and feel a bond with it. That is, product reputation is likely to determine customer feeling state and relationship closeness with the product [38,39]. In other words, the pro-environmental reputation of choosing a certain product will evoke special positive feelings of customers and also create a special customer-product relationship as they feel a bond with it. Taking this into account, the following hypotheses were developed:

**Hypothesis** **7 (H7).** *Pro-environmental reputation has a positive effect on customer-product relationship quality*.

**Hypothesis** **8 (H8).** *Pro-environmental reputation has a positive effect on the feeling of pride*.

### 2.5. Customer-Product Relationship Quality and Its Role

Developing a sturdy relationship quality between a firm and its patron is fundamental for the firm’s success as it reflects the strong affective bonds between two parties [22,41]. Relationship quality is the general assessment of association power between two parties [22]. According to [42], relationship quality is the amount of adequateness of relation to filling the necessities between customer and company. Irrefutably, from the firm’s perspective, endeavors need to center on boosting the relationship strength in that such efforts can play a vital role in building emotional attachment between the firm’s product and its consumers, which eventually results in repeat purchase, enhanced loyalty, and profit increase [22,43]. In the present research, relationship quality is relevant to the overall evaluation of the magnitude of relationship strength between an eco-friendly museum and its customers, which brings either customer positive or negative purchase behaviors to the museum.

Previous empirical studies in marketing and tourism have explored the influence of relationship quality on customer behaviors [22,43,44,45]. When customers select a product/service, relationship quality acts as a crucial role in their decision-making process [44,45]. In the tourism sector, [43] explored the role of relationship quality and tourist behaviors. Their result revealed that customer-product relationship quality plays a vital role in explicating traveler post-purchase decision formation and attitudes. More recently, in the context of cultural tourism, [46] uncovered the role of destination engagement. Their finding indicated that visitor engagement with a destination is a crucial factor in generating traveler revisit and recommendation intentions. [44] investigated luxury cruise travelers’ behaviors. Their empirical finding showed that relationship quality between a cruise tourism product and its consumers significantly influences the consumer loyalty generation process. Consistently, [22], in her recent research, uncovered that brand relationship quality is an imperative factor, affecting customer brand loyalty and positive behaviors for the hotel company. Based on these studies, the strong associations between relationship quality and approach behavioral intentions can be posited as follows:

**Hypothesis** **9 (H9).** *Customer-product relationship quality has a positive effect on sacrifice intention*.

**Hypothesis** **10 (H10).** *Customer-product relationship quality has a positive effect on visit intention*.

**Hypothesis** **11 (H11).** *Customer-product relationship quality has a positive effect on pay intention*.

**Hypothesis** **12 (H12).** *Customer-product relationship quality has a positive effect on WOM intention*.

### 2.6. Feeling of Pride

A substantial amount of evidence successes regarding the essential role of the affective process (e.g., feeling of pride) in explaining customer environment benefiting behaviors [26,30,47,48]. Scholars in diverse tourism contexts stressed the criticality of this affective dimension pertinent to the customer decision-making process [31,49]. Patrons anticipate a certain feeling that they could experience by performing a particular behavior [19,36]. Especially when they get involved in an environment benefiting behavior, they expect to experience a positive affect state [23]. In environmental psychology, individuals’ feeling of pride is the key aspect of such a positive affect state [23,31]. Patrons’ feeling of pride is a crucial emotional process, contributing to boosting the understanding of their pro-environmental intention formation and behavior [19,24,25,36]. Feeling of pride comprises such positive emotional words as accomplished, proud, worthwhile, and confident as its components [26,36].

Scholars in diverse environmental behavior and tourism contexts indicated that linking individuals’ feeling of pride to green behaviors enhances the competence of their conceptual frameworks/models [24,25,26,31]. [24] examined individuals’ travel mode choices. Their result showed that the affective process is a crucial factor influencing individuals’ environmentally responsible travel model selection process. [31] investigated employee green behaviors. Their empirical finding revealed that anticipated pride is a crucial driver of both intrinsically and extrinsically pro-environmental activities. Undoubtedly, patrons often experience a feeling of pride when engaging in pro-environmental consumption behaviors [26]. This affective process can possibly increase the efficacy of a research model for the explanation of approach behaviors for environmentally friendly commodities/services [25,31,36]. Besides, according to [50], feelings of pride exert a meaningful influence on moral obligation/personal norm, which leads to an impact on patrons’ sacrifice intention, visit intention, pay intention, and WOM intention. Referring to the evidence of the above-mentioned studies, we developed the following hypotheses:

**Hypothesis** **13 (H13).** *Feeling of pride has a positive impact on sacrifice intention*.

**Hypothesis** **14 (H14).** *Feeling of pride has a positive impact on visit intention*.

**Hypothesis** **15 (H15).** *Feeling of pride has a positive impact on pay intention*.

**Hypothesis** **16 (H16).** *Feeling of pride has a positive impact on WOM intention*.

### 2.7. Approach Behavioral Intentions

Patrons’ cognitions/perceptions and attitudes associated with environmental issues and problems have been extensively examined by researchers in environmental behavior and tourism over the past decades [48,51]. However, regardless of scholars’ growing acquiescence on the criticality of educating customers about eco-friendly behaviors [52], research that focuses mainly on Patrons’ environmentally sustainable behaviors at a museum is not abundant to date. The term, customer pro-environmental behaviors, indicates any patrons’ actions that conserve the natural environment and minimize the harmful effect of their consumption activities on the environment [53]. Individuals make decisions to sacrifice their benefits, purchase, pay, and recommend an eco-friendly tourism product for environmental preservation [50]. Particularly, eco-conscious patrons often form approach behavioral intentions for the eco-friendly product. [40] identified behavioral intentions as customer willingness to enact a certain attitude toward a specific product. Consistently, in the present study, approach behavioral intentions refer to visitors’ willingness to engage in choices/behaviors for an eco-friendly museum product to preserve the environment (i.e., sacrifice, visit, pay, and WOM intentions). Customers’ eco-friendly consumption behaviors are often under cognitive, social, and affective influences [16,26,50]. Individuals build pro-environmental intentions for such environmentally sustainable products through a convoluted approach to decision-making through cognitive, social, and affective processes [13,26,48,54].

### 2.8. Conceptual Framework and Hypotheses

**Figure 1:**
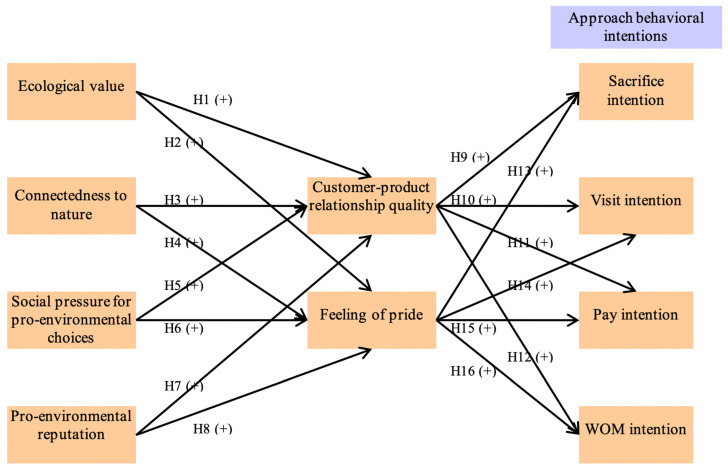
The proposed conceptual framework. H1–H16: hypothesis 1–16.

The proposed conceptual model is exhibited in Figure 1. This proposed framework encompassed ten research variables (i.e., ecological value, connectedness to nature, social pressure for pro-environmental choices, pro-environmental reputation, customer-product relationship quality, feeling of pride, sacrifice intention, visit intention, pay intention, WOM intention). In addition, our research model had 16 research hypotheses linking the variables.

## 3. Method

### 3.1. Measurement Items and Survey Questionnaire

To evaluate study constructs, the measurement items were adopted from previous studies in environmental psychology and tourism literature [10,12,21,28,36,53,55]. Specifically, a total of three items from “not very important” (1) to “very important” (7) were used to assess ecological value (e.g., “Please indicate to what extent the followings are important as a guiding principle in your life—preventing pollution”). For the assessment of connectedness to nature, we utilized four items (e.g., “I think of the natural world as a community to which I belong”). To evaluate pro-environmental reputation, two items were used (e.g., “The overall reputation of an environmentally responsible museum is favorable”). In addition, customer-product relationship quality was evaluated with two items (e.g., “My feeling of belonging to an environmentally responsible museum is strong”). The proud feeling was assessed with three items (i.e., “proud”, “accomplished”, and “worthwhile”). 

Moreover, two items for sacrifice purpose (e.g., “To protect the environment, I would be willing to accept any inconvenience (e.g., recycling, reducing water/energy use, decreasing waste) in an environmentally responsible museum”), two items for visit intention (e.g., “I am willing to visit an environmentally responsible museum in the future”), two items for pay intention (e.g., “I will make an effort to pay for a museum that follows environmental policies and guidelines”), and two items for WOM intention (e.g., “I will say positive things about an environmentally responsible museum”) were utilized. Connectedness to nature, social pressure, pro-environmental reputation, customer-product relationship quality, feeling of pride, and approach behavioral intentions were all evaluated from “strongly disagree” (1) to “strongly agree” (7). The initial survey questionnaire containing these measures was pre-tested with tourism scholars. Based on the pre-test results, a small improvement was made. The questionnaire was then completed with reviews by academic experts. The questionnaire is presented in Appendix A.

### 3.2. Data Collection Procedure and Samples

To obtain research objectives, this research employed a quantitative field survey approach. The survey was carried out at five museums located in Korea’s metropolitan city and its suburban areas. The selected five museums are often considered major museums in Korea as these museums are containing a bigger number of artifacts than other smaller museums in Korea. The five museums were the HXX museum located in Seochogu, the RXX museum in Yongsan-gu, the SXX museum in Junggu, the MXX museum in Jongro, and the MXX museum in Junggu. In addition, these museums also have a good reputation for their exhibits and eco-friendly management and design. The surveyors collected the data mostly in indoor and outdoor rest areas, shops, and cafés/restaurants. In particular, the surveyors approached museum customers and asked their willingness to fill out the survey questionnaire. The surveyors explained the details of the survey purpose and research before receiving the agreement of patrons’ survey participation. The participants completed the survey and returned it on the spot. Through this procedure, the surveyors gained 304 usable responses. These cases were retained for analyzing the data.

## 4. Results

### 4.1. Demographic Profile

Of 304 survey participants, about 54.8% were female visitors, whereas 45.2% were male visitors. The participants were between 19 years old and 76 years old. Their mean age was 30.16 years old. Most participants were highly educated. About 75.8% reported that they have a college degree. In addition, about 15.2% answered that they are graduate degree holders, and 8.9% answered that they are high school graduates or less. Regarding the participants’ annual income level, about 34.6% responded the income between $25,000 and $54,999, followed by the income between $55,000 and $84,999 (24.6%), the income under 24,999 (21.8%), and the income above $100,000 (19.1%). The respondents’ annual museum visit frequency was asked. Their average museum visit was 5.71 times per year.

### 4.2. Confirmatory Factor Analysis and Measurement Quality Assessment

To evaluate the quality of construct measures, confirmatory factor analysis was carried out by using AMOS 22 and SPSS 22 as tools. As presented in Table 1, the measurement model had an excellent model fit (χ^2^ = 345.226, *df* = 207, *p* < 0.001, χ^2^/*df* = 1.668, root mean square error approximation (RMSEA) = 0.047, comparative fit index (CFI) = 0.972, incremental fit index (IFI) = 0.973, and Tuker-Lewis index (TLI) = 0.963). All observed variables were significantly loaded to their associated latent constructs. In addition, a satisfactory level of composite reliability was achieved (ecological value = 0.861, connectedness to nature = 0.809, social pressure = 0.941, pro-environmental reputation = 0.931, customer-product relationship quality = 0.901, feeling of pride = 0.884, sacrifice intention = 0.690, visit intention = 0.853, pay intention = 0.906, and WOM intention = 0.930), which demonstrated the internal uniformity of the within-construct items [56]. Moreover, a satisfactory level of average variance extracted values were obtained. The values (ecological value = 0.674, connectedness to nature = 0.519, social pressure = 0.888, pro-environmental reputation = 0.871, customer-product relationship quality = 0.821, feeling of pride = 0.646, sacrifice intention = 0.527, visit intention = 0.744, pay intention = 0.828, and WOM intention = 0.869) surpassed the minimum threshold of 0.50 [56]. As exhibited in Table 1, these values were also higher than between-construct correlations. Hence, the discriminant validity of construct measures was demonstrated.

### 4.3. Structural Equation Modeling and Hypotheses Testing

**Figure 2:**
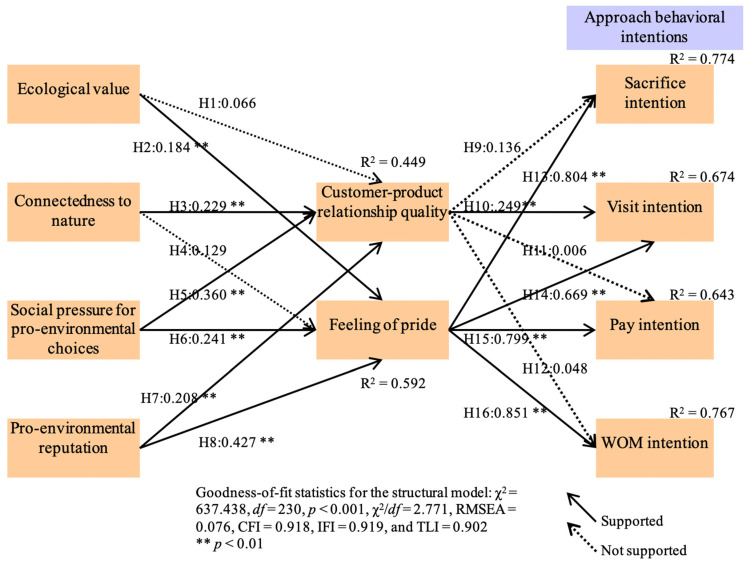
The structural model results. H1–H16: hypothesis 1–16; RMSEA: root mean square residual; CFI: comparative fit index; IFI: incremental fit index; TLI: Tuker-Lewis index.

Evaluating the hypothesized theoretical framework, structural equation modeling was employed. As depicted in Figure 2 and Table 2, the model included an excellent model fit (χ^2^ = 637.438, *df* = 230, *p* < 0.001, χ^2^/*df* = 2.771, RMSEA = 0.076, CFI = 0.918, IFI = 0.919, and TLI = 0.902). The proposed model had a satisfactory forecasting power for customer approach behavioral intentions. In particular, about 77.4% and 67.4% of the variance in sacrifice intention and visit intention were explained by their antecedents, respectively. Approximately 64.3% and 76.7% of the variance in pay intention and WOM intention were explained by their predictors, respectively. In addition, ecological value, connectedness to nature, social pressure, and pro-environmental reputation accounted for about 44.9% and 59.2% of the variance in relationship quality and feeling of pride, respectively.

The hypothesized impact of ecological value was examined. Results showed that ecological value had a positive feeling of pride (β = 0.184, *p* < 0.01). Yet, its effect on relationship quality was not significant (β = 0.066, *p* > 0.05). Therefore, Hypothesis 1 was not supported, while Hypothesis 2 was supported. The proposed impact of connectedness to nature was assessed. Our findings showed that connectedness to nature exerted a positive impact on customer-product relationship quality (β = 0.229, *p* < 0.01), but its impact on feeling of pride was not significant (β = 0.129, *p* > 0.05). Hence, Hypothesis 3 was supported, whereas Hypothesis 4 was not supported. The hypothesized influence of social pressure was evaluated. Predictably, social pressure for pro-environmental choices contained a positive effect on relationship quality (β = 0.360, *p* < 0.01) and feeling of pride (β = 0.241, *p* < 0.01). This result approved Hypotheses 5 and 6 as valid. The proposed effect of pro-environmental reputation was evaluated. Our findings revealed that pro-environmental reputation had a positive impact on relationship quality (β = 0.208, *p* < 0.01) and feeling of pride (β = 0.427, *p* < 0.01). Therefore, Hypotheses 7 and 8 were approved as valid.

Subsequently, the association between customer-product relationship quality and approach intentions was evaluated. Results revealed that relationship quality had a positive influence on visit intention (β = 0.249, *p* < 0.01). However, its influence on sacrifice intention (β = 0.136, *p* > 0.05), pay intention (β = 0.006, *p* > 0.05), and WOM intention (β = 0.048, *p* > 0.05) was not significant. Hence, while Hypothesis 10 was supported, Hypotheses 9, 11, and 12 were not supported. The relationship between the feeling of pride and approach intentions was examined. As expected, feeling of pride brought a positive effect on visit intention (β = 0.804, *p* < 0.01), sacrifice intention (β = 0.669, *p* < 0.01), pay intention (β = 0.799, *p* < 0.01), and WOM intention (β = 0.851, *p* < 0.01). This result approved Hypotheses 13, 14, 15, and 16 as valid.

### 4.4. Mediating Effect Assessment

The indirect influence of the study variables was examined. Table 2 includes the details about the indirect impact testing using a bootstrap method. Our result showed that ecological value (β = 0.157, *p* < 0.01), social pressure for pro-environmental choices (β = 0.242, *p* < 0.01), and pro-environmental reputation (β = 0.372, *p* < 0.01) had a significant and positive indirect effect on sacrifice intention. Visit intention was also under the meaningful indirect effect of ecological value (β = 0.139, *p* < 0.05), connectedness to nature (β = 0.143, *p* < 0.05), social pressure (β = 0.250, *p* < 0.05), and pro-environmental reputation (β = 0.337, *p* < 0.01). In addition, our findings revealed that ecological value (β = 0.147, *p* < 0.01), social pressure (β = 0.194, *p* < 0.05), and pro-environmental reputation (β = 0.342, *p* < 0.01) exerted a meaningful indirect impact on pay intention. Moreover, WOM intention was under the significant and positive indirect influence of ecological value (β = 0.160, *p* < 0.05), social pressure (β = 0.222, *p* < 0.01), and pro-environmental reputation (β = 0.374, *p* < 0.01). Overall, this result implied that both customer-product relationship quality and feeling of pride played a crucial mediator role within the hypothesized conceptual framework.

## 5. Discussion

The present study apparently expanded museum academics’ and practitioners’ understanding of customer approach intention formation and behavior for environmentally responsible museums. In particular, our research apparently uncovered the impact of ecological value, connectedness to nature, social pressure, and pro-environmental reputation on environment-friendly decision-making procedures among museum customers. Additionally, this research clearly explored the role of customer-product relationship quality and feeling of pride as direct contributors to approach intentions and as mediators. Our study also discovered that customer feeling of pride was a salient driver of sacrifice, visit, pay, and WOM intentions. While social pressure was the main contributor to increasing relationship quality, pro-environmental reputation was a major trigger of customer feeling of price. The proposed theoretical framework showed an adequate level of ability for predicting customer pro-environmental intentions to sacrifice, visit, pay, and practice WOM behaviors.

As far as we know, our study was the first study that analyzed the process of generating the multiple dimensions of eco-friendly approach intentions among museum customers. In addition, this research was the first to link ecological value, connectedness to nature, social pressure, pro-environmental reputation, customer-product relationship quality, and feeling of pride for building approach intentions in a successful manner. To our knowledge, the present research was one of the few studies that thoroughly investigated museum customer pro-environmental decision formation by incorporating the mediating effect of customer-product relationship quality and feeling of pride. Taken as a whole, moving one step further, our research successfully developed a sturdy theoretical framework that effectively accounts for customer sacrifice, visit, pay, and WOM decisions for environmentally friendly museum products. 

The findings of this study revealed that social pressure and pro-environmental reputation among cognitive factors were especially vital concepts, contributing to increasing customer-product quality and feeling of pride, respectively. From the theoretical aspect, this result offered the crucial theorization that enhancing the level of customer social pressure for pro-environmental choices and boosting the level of pro-environmental reputation of a museum increase relationship quality and pride feeling, eventually generating customer eco-friendly intentions for the museum. Museum practitioners, therefore, should inform their existing and potential customers of the various types of environmental preservation efforts of eco-friendly museums (e.g., greywater use, recycling, energy-saving, green management, green design of a building, natural light use) through many communication channels (e.g., Social Network Service, media). This endeavor could ultimately result in museum customers’ increased social pressure for pro-environmental choices and in the high eco-friendly reputation of the museum. 

Such an affective element as a feeling of pride is essential in justifying one’s pro-environmental behavior [24,25,26]. Yet, the pride of oneself and its importance have been overlooked in extant socio-psychological theories in the social/environmental behavior context (e.g., norm activation theory, value-belief-norm model, the theory of green purchase behaviors) [13,20]. This affective factor was uncovered as a fundamental driving force of museum visitor pro-environmental intentions for eco-friendly museums in our conceptual framework. A strong prediction power for customer pro-environmental intentions under our hypothesized theoretical model encompassing the feeling of pride was sturdily supported. Particularly, concerning the function of the feeling of pride, this affective variable, mostly formed based on ecological value and pro-environmental reputation, acted as a salient determinant of sacrifice, visit, pay, and WOM decisions among museum customers. This crucial result informed museum academics and operators that engendering customer feeling of “proud”, “accomplished”, and “worthwhile” related to museum product consumption is an efficient way to maximize customer approach decisions to visit an eco-friendly museum, say positive things about it (or recommend it to others), pay for it, and accept any inconveniences for it (e.g., recycling, saving water/energy, minimizing waste). 

As demonstrated in this research, customer-product relationship quality exerts a significant effect on visit intention, and feeling of pride exerts a significant impact on all approach intentions. On the basis of our findings, it was also apparent that customer-product relationship quality and feeling of pride mediated the influence of their antecedents. This means that if customer-product relationship quality and feeling of pride are present, the effect of ecological value, connectedness to nature, social pressure, and pro-environmental reputation on approach behavioral intentions can be maximized. Our result provided museum academics and professionals the essential knowledge that involving these two constructs and their mediating influence into the theoretical model is of importance for the clear understanding of the role of ecological value, connectedness to nature, social pressure, and reputation. Museum researchers and practitioners should recognize the mediating nature of relationship quality and feeling of pride when developing a conceptual model and broadening the conceptual frameworks for pro-environmental intentions/behaviors in the extant literature. Our findings moreover informed museum professionals of what dealing with these mediating factors is imperative means to obtain full advantage of cognitive variables (ecological value, connectedness to nature, social pressure, and reputation) for ascending museum customer positive behavioral intentions.

This research has a few limitations. Firstly, the present research focused on investigating the cognitive and affective drivers of pro-environmental decision-making procedure. Yet, researches in environmental psychology and consumer behavior also stressed the essential role of normative/moral factors and their considerable influence on eco-friendly intention generation (e.g., [29,32,57]). This normative/moral variable and its potential impact should be considered for future studies. Second, several studies asserted that social psychological theories with self-interest motives (e.g., the theory of planned behavior, a model of goal-directed behavior) are also effective in explaining customer environmentally responsible behaviors (e.g., [10,36,58]). For future research, expanding the proposed model by integrating key concepts within such theories is recommended for the increase in its comprehensiveness and prediction power. Lastly, instead of using a Likert scale of function value in future research, the willingness to pay (WTP) of consumers for environment-friendly museums may be a great measure.

## Figures and Tables

**Table 1:** The measurement model assessment results and correlations (*n* = 304).

Measurement Item	(1)	(2)	(3)	(4)	(5)	(6)	(7)	(8)	(9)	(10)	β
(1) Ecological value	1.000	–	–	–	–	–	–	–	–	–	0.787
Ecological value2											0.800
Ecological value3											0.875
(2) Connectedness to nature	0.457 ^a^	1.000	–	–	–	–	–	–	–	–	0.556
Connectedness to nature2	(0.297) ^b^										0.787
Connectedness to nature3											0.726
Connectedness to nature4											0.788
(3) Social pressure for pro-environmental choices	0.254	0.311	1.000	–	–	–	–	–	–	–	0.981
Social pressure for pro-environmental choices2	(0.065)	(0.097)									0.902
(4) Pro-environmental reputation	0.443	0.459	0.383	1.000	–	–	–	–	–	–	0.937
Pro-environmental reputation2	(0.196)	(0.211)	(0.147)								0.929
(5) Customer-product relationship quality	0.370	0.419	0.511	0.464	1.000	–	–	–	–	–	0.957
Customer-product relationship quality2	(0.137)	(0.176)	(0.261)	(0.215)							0.852
(6) Feeling of pride	0.318	0.262	0.390	0.448	0.423	1.000	–	–	–	–	0.901
Feeling of pride2	(0.101)	(0.069)	(0.152)	(0.201)	(0.179)						0.707
Feeling of pride3											0.792
(7) Sacrifice intention	0.359	0.364	0.287	0.457	0.461	0.432	1.000	–	–	–	0.724
Sacrifice intention2	(0.129)	(0.132)	(0.082)	(0.209)	(0.213)	(0.178)					0.728
(8) Visit intention	0.396	0.411	0.418	0.469	0.539	0.456	0.680	1.000	–	–	0.872
Visit intention2	(0.157)	(0.169)	(0.175)	(0.220)	(0.291)	(0.208)	(0.462)				0.853
(9) Pay intention	0.337	0.381	0.335	0.504	0.405	0.412	0.640	0.599	1.000	–	0.903
Pay intention2	(0.114)	(0.145)	(0.112)	(0.254)	(0.164)	(0.170)	(0.410)	(0.359)			0.917
(10) WOM intention	0.495	0.460	0.408	0.617	0.461	0.526	0.607	0.617	0.699	1.000	0.952
WOM intention	(0.245)	(0.212)	(0.166)	(0.381)	(0.213)	(0.277)	(0.368)	(0.381)	(0.489)		0.912
Mean	5.507	5.156	4.433	5.341	4.290	4.964	4.648	4.776	4.827	5.076	
(standard deviation)	(1.006)	(0.923)	(1.174)	(1.115)	(1.236)	(0.992)	(1.103)	(1.078)	(1.183)	(1.140)	
Composite reliability	0.861	0.809	0.941	0.931	0.901	0.844	0.690	0.853	0.906	0.930	
(average variance extracted)	(0.674)	(0.519)	(0.888)	(0.871)	(0.821)	(0.646)	(0.527)	(0.744)	(0.828)	(0.869)	

Note. Goodness-of-fit statistics for the measurement model: χ^2^ = 345.226, *df* = 207, *p* < 0.001, χ^2^/*df* = 1.668, RMSEA = 0.047, CFI = 0.972, IFI = 0.973, and TLI = 0.963. ^a^ Correlations between the constructs; ^b^ Squared correlations.

**Table 2:** The measurement model assessment results and correlations (*n* = 304).

Hypothesis	Paths			β	*t*-Values	
H1	Ecological value	→	Customer-product relationship quality	0.066	0.968	
H2	Ecological value	→	Feeling of pride	0.184	2.909 **	
H3	Connectedness to nature	→	Customer-product relationship quality	0.229	3.001 **	
H4	Connectedness to nature	→	Feeling of pride	0.129	1.880	
H5	Social pressure for pro-environmental choices	→	Customer-product relationship quality	0.360	6.374 **	
H6	Social pressure for pro-environmental choices	→	Feeling of pride	0.241	4.353 **	
H7	Pro-environmental reputation	→	Customer-product relationship quality	0.208	3.070 **	
H8	Pro-environmental reputation	→	Feeling of pride	0.427	6.013 **	
H9	Customer-product relationship quality	→	Sacrifice intention	0.136	1.807	
H10	Customer-product relationship quality	→	Visit intention	0.249	3.860 **	
H11	Customer=product relationship quality	→	Pay intention	0.006	0.096	
H12	Customer-product relationship quality	→	WOM intention	0.048	0.824	
H13	Feeling of pride	→	Sacrifice intention	0.804	7.605 **	
H14	Feeling of pride	→	Visit intention	0.669	7.817 **	
H15	Feeling of pride	→	Pay intention	0.799	8.472 **	
H16	Feeling of pride	→	WOM intention	0.851	9.440 **	
Indirect effect on sacrifice intention:		Indirect effect on visit intention:		Indirect effect on pay intention:	Indirect effect on WOM intention:	Total Variance Explained
β ecological value = 0.157 ** β connectedness to nature = 0.135 β social pressure = 0.242 ** β pro-environmental reputation = 0.372 **		β ecological value = 0.139 ** β connectedness to nature = 0.143 * β social pressure = 0.250 * β pro-environmental reputation = 0.337 **		β ecological value = 0.147 ** β connectedness to nature = 0.104 * β social pressure = 0.194 * β pro-environemental reputation = 0.342 **	β ecological value = 0.160 * β connectedness to narure = 0.121 β social pressure = 0.222 ** β pro-environmental reputation = 0.347 **	R^2^ (customer product relationship quality) = 0.449 R^2^ (feeling of pride) = 0.592 R^2^ (sacrifice intention) = 0.774 R^2^ (visit intention) = 0.674 R^2^ (pay intention) = 0.643 R^2^ (WOM intention) = 0.767

Note. Goodness-of-fit statistics for the structural model: χ^2^ = 637.438, *df* = 230, *p* < 0.001, χ^2^ /*df* = 2.771, RMSEA = 0.076, CFI = 0.918, IFI = 0.919, and TLI = 0.902. * *p* < 0.05 and ** *p* < 0.01.

## Data Availability

The dataset used in this research are available upon request from the corresponding author. The data are not publicly available due to restrictions i.e., privacy or ethical.

## Appendix A

**Ecological value** [16]

*Please indicate to what extent the followings are important as a guiding principle in your life*.

Preventing pollution

Unity with nature

Protecting the environment

**Connectedness to nature** [44]

I often feel a sense of oneness with the natural world around me.

I think of the natural world as a community to which I belong.

I recognize and appreciate the intelligence of other living organisms.

I often feel like I am only a small part of the natural world around me.

**Social pressure for pro-environmental choices** [23]

Most people who are important to me think I should visit an environmentally responsible museum instead of a conventional museum.

Most people who are important to me would want me to visit an environmentally responsible museum instead of a conventional museum.

**Pro-environmental reputation** [20]

The overall reputation of an environmentally responsible museum is favorable.

Overall, an environmentally responsible museum has a good reputation.

**Customer-product relationship quality** [22]

My level of emotional attachment to an environmentally responsible museum is high.

My feeling of belonging to an environmentally responsible museum is strong.

**Feeling of pride** [50]

*The image that you are visiting an environmentally responsible museum, which minimizes its negative impact on the environment. How would you feel?*

Proud

Accomplished

Worthwhile

**Sacrifice intention** [50]

To protect the environment, I would be willing to pay more for an environmentally responsible museum.

To protect the environment, I would be willing to accept any inconvenience (e.g., recycling, reducing water/energy use, decreasing waste) in an environmentally responsible museum.

**Visit intention** [36]

I am willing to visit an environmentally responsible museum in the future.

I plan to visit an environmentally responsible museum in the future.

**Pay intention** [10]

I am willing to pay conventional-museum prices for an environmentally responsible museum.

I will make an effort to pay for a museum that follows environmental policies and guidelines.

**WOM intention** [10]

I will encourage my family, friends, and relatives to visit an environmentally responsible museum.

I will say positive things about an environmentally responsible museum.
